# Strong and recurring seasonality revealed within stream diatom assemblages

**DOI:** 10.1038/s41598-018-37831-w

**Published:** 2019-03-01

**Authors:** M. A. Snell, P. A. Barker, B. W. J. Surridge, C. McW. H. Benskin, N. Barber, S. M. Reaney, W. Tych, D. Mindham, A. R. G. Large, S. Burke, P. M. Haygarth

**Affiliations:** 1 Lancaster Environment Centre, Lancaster University, Lancaster, LA1 4YQ UK; 2 Agri-Food and Biosciences Institute, Newforge Lane, Belfast, BT9 5PQ UK; 3 Department of Geography, Durham University, Durham, DH1 3LE UK; 4 School of Geography, Politics and Sociology, Newcastle University, Newcastle upon Tyne, NE1 7RU UK; 5 British Geological Survey, Environmental Science Centre, Nicker Hill, Keyworth, Nottingham, NG12 5GG UK

## Abstract

Improving stream water quality in agricultural landscapes is an ecological priority and a legislative duty for many governments. Ecosystem health can be effectively characterised by organisms sensitive to water quality changes such as diatoms, single-celled algae that are a ubiquitous component of stream benthos. Diatoms respond within daily timescales to variables including light, temperature, nutrient availability and flow conditions that result from weather and land use characteristics. However, little consideration has been given to the ecological dynamics of diatoms through repeated seasonal cycles when assessing trajectories of stream function, even in catchments actively managed to reduce human pressures. Here, six years of monthly diatom samples from three independent streams, each receiving differing levels of diffuse agricultural pollution, reveal robust and repeated seasonal variation. Predicted seasonal changes in climate-related variables and anticipated ecological impacts must be fully captured in future ecological and water quality assessments, if the apparent resistance of stream ecosystems to pollution mitigation measures is to be better understood.

## Introduction

In the context of a changing climate and agricultural intensification, it is recognised that freshwater ecosystems are vulnerable to multiple anthropogenic stressors, including excess sediment and nutrient delivery, whilst also being modulated by climate-dependent flow, water temperature and event-driven transfers from catchments^1–3^. Low-order streams comprise the headwaters of river networks and drain a significant proportion of the UK’s agricultural land. Such streams are central to the functioning of all river networks, forming important corridors linking terrestrial and aquatic ecosystems. These reaches are especially vulnerable to stress as their small channel size relative to catchment area makes them poorly buffered to changes in external climatic, physicochemical and energetic factors^4–6^. However, low-order streams play an important role in regulating downstream processes and, ultimately, the quality of coastal or other receiving waters^7,8^.

Ecosystem health is assessed using ecological surveys of stream benthos to identify anthropogenic impacts and trajectories of change^9–11^. Within Europe, biological monitoring has been formalised through the Water Framework Directive (WFD)^12^. This legislation requires an assessment of ecological health, reported as an Ecological Quality Ratio (EQR) which represents the value of an observed biological parameter to that expected under minimally-impaired conditions characteristic of the waterbody type under consideration^13^. The EQR can vary between values indicative of ‘high’ to ‘bad’ status, with intermediate values indicative of ‘good’, ‘moderate’ or ‘poor’ status. The EQR is based on specific groups of organisms, including macrophytes, fish, macroinvertebrates and diatoms, and informs subsequent development of restorative measures to reduce significant anthropogenic pressures. Full consideration of organism colonisation, resilience to high-energy events and seasonal controls is needed to produce robust biological datasets that capture ecosystem function and variability. Failure to consider this temporal dynamic could bias inferences drawn from ecological assessment and contribute to poorly-targeted mitigation within catchments, potentially incurring significant economic cost.

The incorporation of weather-related factors within monitoring programmes, such as solar radiation, temperature and rainfall that are descriptive of multi-annual seasonal trends, is critical because future climate scenarios are anticipated to have both direct impacts on stream ecosystems and indirect effects mediated via wider catchment processes. For example, the export of phosphorus (P) from agricultural land to waterbodies is predicted to increase in the future, independent of land use change^14^. Moreover, climate scenarios emphasise an acute increase in the extremes of seasonal cycles with warmer, wetter, winters and hotter, drier, summers in parts of NW Europe^15^. However, the relative contribution of weather, catchment land use and reach-scale processes on benthic stream ecology has not been rigorously assessed to date via multi-annual, high-resolution *in situ* environmental monitoring programmes. This is particularly true within highly dynamic low-order streams. We hypothesise that robust and recurrent seasonal controls on stream ecosystems could overwhelm, or at least partially mask, efforts to reduce the effects of anthropogenic stress on streams. Here we present six full years of monthly diatom data from three separate intensively instrumented tributaries of the River Eden, NW England. For the first time, these data enable us to evaluate the resilience and recurrence of seasonal patterns in benthic diatom assemblages. These diatom data are evaluated alongside high-resolution environmental monitoring data to determine the relative discrete and combined contributions of land use, the seasonality of precipitation, temperature and light, and in-stream environmental variables that combine to define the niche experienced by benthic diatoms.

## Results

**Figure 1:**
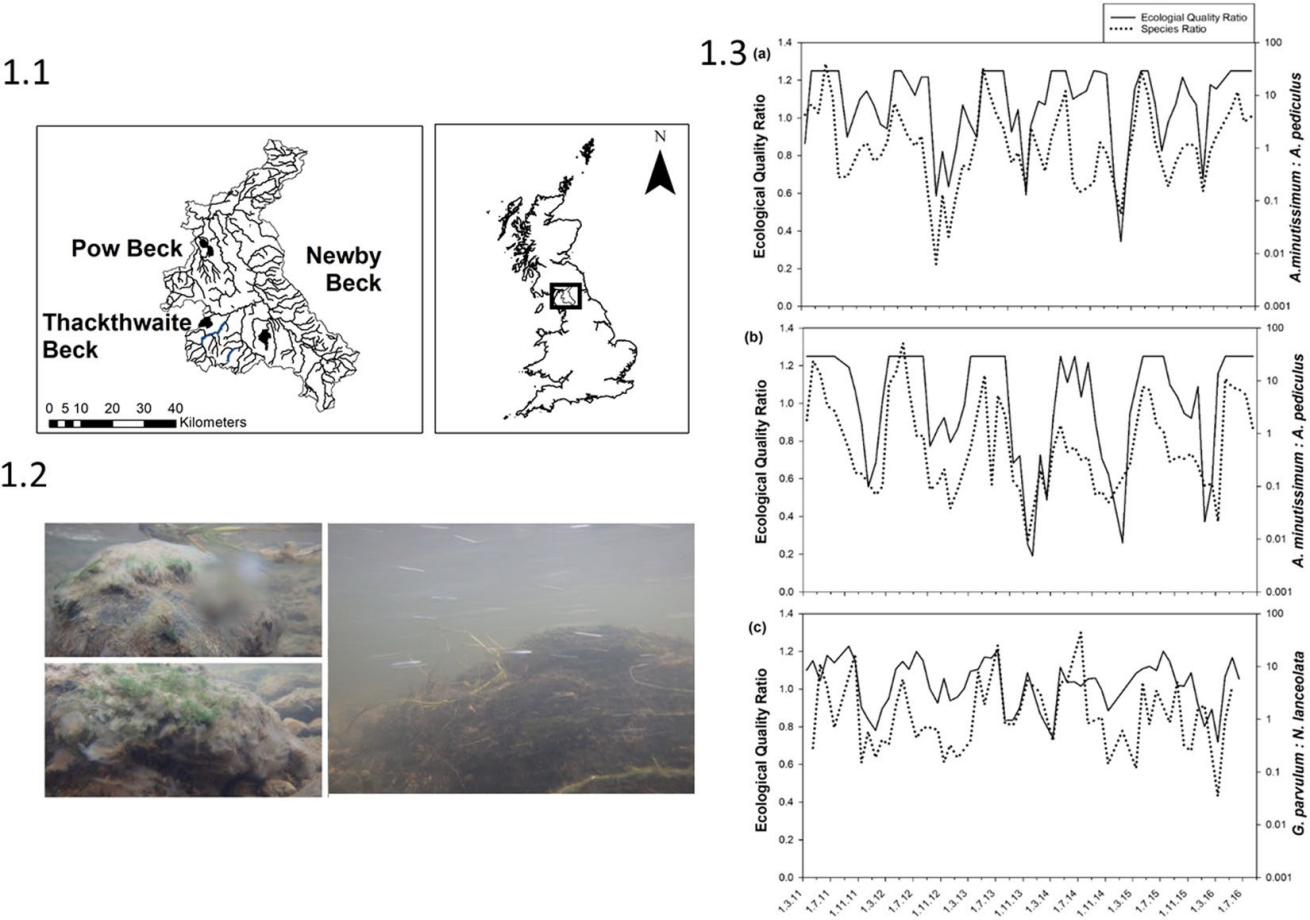
1.1) Newby, Thackthwaite and Pow Beck catchments of the River Eden, NW England. Crown Copyright/database right 2014. An Ordnance Survey/EDINA supplied service; 1.2) Images of the diatom biofilm assemblages *in situ* at Newby Beck November 2015; 1.3) (**a**) Monthly Ecological Quality Ratio (EQR: a diatom-based WFD community metric which measures ecological status) for Pow Beck (solid line) and ratio of *Achnanthidium minutissimum* to *Amphora pediculus* (dashed line) (**b**) Monthly EQR for Newby Beck (solid line) and ratio of *Achnanthidium minutissimum* to *Amphora pediculus* (dashed line) and (**c**) Monthly EQR for Thackthwaite Beck (solid line) and ratio of *Gomphonema parvulum* and *Navicula lanceolata* (dashed line) from March 2011 to August 2016. EQR status boundaries are: High/good = 0.8; Good/moderate = 0.6; Moderate/poor = 0.4; Poor/bad = 0.2 (DARLEQ 2).

Distinct and recurring summer and winter diatom assemblages and EQR values are revealed in the monthly data from all three sub-catchments (Fig. 1.3). The greatest range in ecological status was observed within Newby Beck where EQR values varied between those indicative of ‘high’ to ‘bad’ water quality. In contrast, within Thackthwaite Beck which is subject to the lowest intensity of agricultural production, EQR values cycled within the ‘high’ to ‘good’ status classes through all seasons. In all three sites, the EQR indicates much better water quality in spring/summer compared to autumn/winter (Fig. 1.3a–c). Strong stability of diatom assemblages within Pow Beck (Fig. 1.3a) and Newby Beck (Fig. 1.3b) is evidenced through the dominance, in terms of relative abundance determined via valve counts, of two key species; *Achnanthidium minutissimum* and *Amphora pediculus*. Periods of improved water quality, as determined by the EQR, are associated with an increase in the ratio of *A*. *minutissimum* to *A*. *pediculus*, a pattern that is repeated through all six annual cycles. *A*. *minutissimum* is a small non-colonial pioneer species (length 15 µm, width 3.5 µm)^16^, which colonises and reproduces rapidly^17^ and is often abundant under low phosphorus availability^18^. *A*. *pediculus*, while also a small pioneer species adapted to dynamic discharge conditions (length 11.5 µm, width 3 µm)^16^, favours environments in which nutrient availability is enhanced^19^. At Thackthwaite Beck, the least impacted of the three sites in terms of nutrient enrichment, *A*. *minutissimum* dominated throughout each year and increased in relative abundance during spring and summer. At this site *A*. *minutissimum* was almost seven times more abundant than the next two most abundant species; *Gomphonema parvulum* and *Navicula lanceolata*. Nevertheless, these latter two species also display repeated seasonal patterns throughout the time series, with the motile guild species *N*. *lanceolata* increasing in dominance under autumn/winter conditions (Fig. 1c) suggesting that higher current velocities and nutrient concentrations could be behind this temporal pattern. The dominance of pioneer species across our dataset suggests that these diatom assemblages are kept in a dynamic equilibrium state throughout the annual cycle. Further functional responses are found in Thackthwaite Beck, where repeated switching in dominance between *G*. *parvulum* and *N*. *lanceolata* represents the temporal interaction between a high-profile, larger species characteristic of nutrient enrichment (*G*. *parvulum)*, and species indicative of the motile guild that are better adapted to high flow-low nutrient availability conditions (*N*. *lanceolate)*. Although different species composition was found between the three independent streams, the same co-aligned seasonal pattern of species change was observed.

**Figure 2:**
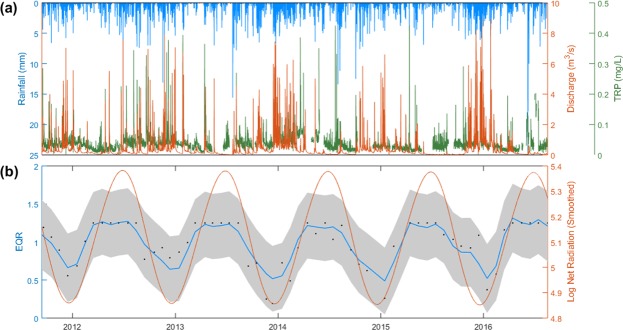
Flow and water chemistry data for the period from 14 September 2011 to 16 August 2016 for Newby Beck: (**a**) Hourly rainfall, discharge and Total Reactive Phosphorus (TRP); (**b**) Monthly diatom EQR and Log Net Radiation. ASDHR model fit with 95% confidence interval explaining over 80% of data variance, and showing the strength of seasonal character of the process. Log Net Radiation Smoothed is the natural log of Net Radiation prior to using DHR-demonstrating Net Radiation seasonality + smoothing.

Environmental controls on species composition were investigated using a 5-year subset of data from Newby Beck, comprising the most complete flow and water chemistry data (Fig. 2a). These high frequency *in situ* measurements reveal a strong relationship between weather-related variables (including rainfall, radiation and temperature), discharge conditions, and nutrient and sediment concentrations^14,20^. The cyclical nature of the monthly diatom data is strongly supported by ASDHR results (Arbitrary Sampled Dynamic Harmonic Regression; Fig. 2b, showing the data against the model with its 95% confidence band). This method allows estimation of periodic or seasonal signal components in irregularly-sampled time-series data, providing both an estimate of the periodic components themselves as well as their uncertainty estimates (see Methods). The model explained 82% of data variance (R^2^ = 0.82) in the EQR metric and the ANOVA F-test was significant at *p* = 5.2 ∙ 10^−10^.

**Figure 3:**
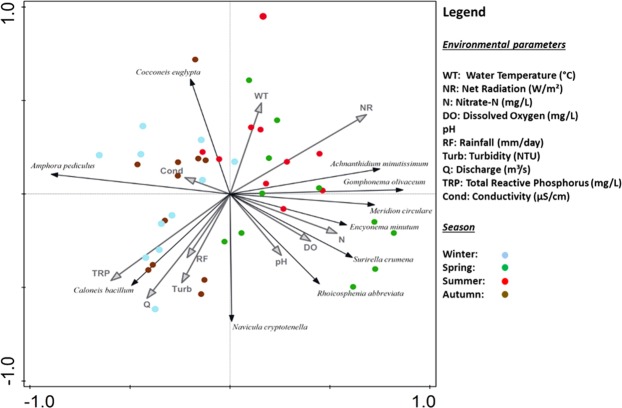
Principal Component Analysis with monthly diatom species scores limited to 10 by fit. Environmental variables averaged over an antecedent window of 21 days prior to biological sample collection. Explained cumulative variation is 34.3% with an adjusted explained variation of 26.1%.

Diatom assemblages typically develop over 1–3 weeks^20^, so the mean value of environmental variables (water temperature, net radiation, nitrate-N, dissolved oxygen, pH, rainfall, turbidity, discharge, total reactive phosphorus and conductivity) for the 21 days prior to diatom sampling were used to explore species-environment interactions. Spring and summer diatom samples scored highly against Principal Component Analysis (PCA) axis 1 with an eigenvalue of 0.2, demonstrating the primary importance of light. These samples were characterised by the presence of *Gomphonema olivaceum* and *A*. *minutissimum*. *G*. *olivaceum* is a high-profile guild species indicating resource-unlimited but disturbance-stress conditions, while *A*. *minutissimum*, the dominant species in terms of relative abundance as determined by cell counts, is a low-profile guild species that is comparatively resistant to physical disturbance. Under spring/summer conditions, water temperature and net radiation, dissolved oxygen and nitrogen (N) availability are key drivers of the diatom assemblage composition. Variability in spring/summer conditions was indicated through low-profile (*Meridion circulare*, *Rhoicosphenia abbreviata*), high-profile (*Encyonema minutum*) and motile (*Surirella crumena*) guild species. In contrast, the PCA demonstrates that winter and autumn diatom samples are associated with in-stream conditions, primarily turbidity and discharge, precipitation-related climate variations and phosphorus resource availability, which all plot in the lower left quadrant of Fig. 3. The percentage of *A*. *minutissimum* is inversely related to *A*. *pediculus*, the dominant pioneer species under rainfall-driven discharge-phosphorus winter conditions^14^. The presence of low-profile (*Cocconeis euglypta*) and motile (*Navicula cryptotenella* and *Caloneis bacillum*) guild species along with *A*. *pediculus* indicates the importance of variables occurring under winter conditions. Seasonality and nutrient resource availability are important in determining contrasting summer and winter ecological states, with instream variables of conductivity, water temperature and pH switching in relative importance as drivers of diatom assemblage composition between seasons.

## Discussion

These three independent and contrasting sub-catchments of the River Eden, characterised by different levels of agricultural intensity, demonstrate robust and recurring seasonal patterns in stream benthic diatom assemblages over a six-year period. The resilience of the assemblage at this seasonal scale is greater than shown previously^21,22^ offering species-environment insights for catchment management programmes. Moreover, intensive instrumentation in these catchments has allowed antecedent environmental conditions occurring during biofilm formation to be captured^20^. Our study demonstrates the crucial temporal perspective that is required to understand ecosystem variability, function and response to seasonality at inter- and intra-annual scales. The long-term diatom time-series displays a dynamic community of pioneer species, implying a constant resetting of the benthic community constrained within characteristic seasonal equilibria. The diatom taxa reflect the impact of both nutrient and stream conditions with a seasonal signature emerging through changes in relative species abundance. Changes in these underlying drivers, both anthropogenic (e.g. discharge associated changes in phosphorus concentration) and weather-related (water temperature, net radiation, and rainfall), combine to shift the ecosystem into clearly-defined alternative states under winter and summer conditions. Community variation left unexplained in the analysis can potentially be attributed to variables not measured in this study, including the influence of grazing^23^. To increase our ability to predict ecological responses to changes in regional weather and climate patterns, it is necessary for future studies to integrate long-term, empirical survey data with models and manipulative experiments.

Multiple seasonal trends at regional scale provide a dynamic baseline against which land-use decisions are made, and this baseline itself is expected to change in the future. For example, projections for the study catchment region suggest a rise in mean annual temperature between 2.5 °C to 3.3 °C by the 2070 s under a ‘medium’ emissions scenario. The anticipated outcome is that more intense seasonal changes will be observed in rainfall, with drier summer and wetter winter conditions expected^15^. Across seasonal and multi-annual scales, direct weather variables (temperature, light) are the dominant factors dictating diatom assemblage, with seasonally-delivered nutrients providing further temporally-mediated regulation of diatom assemblages. An important consequence is that future climate changes could undermine catchment remediation programmes whose ‘success’ is typically measured over the short-term. Furthermore, current ecological monitoring frameworks are based largely on detecting responses to organic pollution under spring and autumn conditions. These programmes give little consideration to seasonal characteristics of diatom assemblages and will not capture this shifting seasonal baseline, nor how the health of a stream ecosystem over a full annual cycle may respond to land management strategies. Given climate predictions and suggested requirements for agricultural land-use change to mitigate phosphorus export^24^, current assessments of ‘ecological status’ will increasingly become obsolete over time, as the baseline drifts to new warmer and wetter conditions. The different components of seasonal change (e.g. temperature, hydrology and atmospheric composition) not only affect multiple levels of biological organisation, but may also interact with other facets of anthropogenic stress to which rivers are exposed^25–27^. It is critical that future research addresses these potentially important synergies underpinned by a clear understanding of climate forcing and trajectories. Given the evidence presented here showing a strong and recurring seasonality in benthic diatoms in stream ecosystems, understanding future ecological variability is important to inform current and future conservation strategies. Capturing this dynamic will ensure that ecological function is maintained across all seasons, as well as allowing for better predictive models of how freshwater systems may respond to management under increasingly contrasting seasonal conditions.

## Methods

### Description of study sites

This study presents data for three low-order streams (Fig. 1 (1.1)); Newby Beck (Strahler second order: OSGB NY-59957,21249) which drains 12.5 km^2^ of the headwaters of the Morland catchment, Pow Beck (Strahler third order: OSBG NY-38685,50074) which drains an area of 10.5 km^2^ and Thackthwaite Beck (Strahler second order: OSBG NY-41190-25320) which drains 10.2 km^2^ of the headwaters of the Dacre Beck catchment, within the larger River Eden catchment, NW England. The three sub-catchments form part of the River Eden UK Demonstration Test Catchment (DTC) programme, a catchment-scale research platform funded by the Department for the Environment, Food and Rural Affairs (2009–2018). Differences in bedrock, superficial geology, land use, rainfall and water quality characteristics are found among the three sub-catchments^28^. Briefly, Pow Beck, with sandstone bedrock, supports intensive agricultural land use practice giving rise to higher diffuse N and P pressures within the catchment. Thackthwaite Beck, lying on hard rock of volcanic andesite sheets, is a flashy, oligotrophic, upland catchment. Newby Beck drains exposed steeply dipping, fractured carboniferous limestone, shale and sandstone units. Having improved grassland, with rough grazing and arable land as the predominant land use, Newby Beck is exposed to intermediate diffuse pollution pressures in comparison to the Pow Beck and Thackthwaite Beck catchments. All three catchments have a superficial layer of post-glacial till, separating much of the surface hydrological response from the direct influence of the bedrock geology.

### Diatom preparation and identification

Diatoms were collected mid-monthly from March 2011 until August 2016 for all three streams. On each sampling occasion, five representative submerged cobbles were selected from typical riffle areas. Cobbles were scraped with a hard bristle brush and the diatom suspension collected. Samples were processed using 30% hydrogen peroxide and permanent slides were prepared using Naphrax^29^. 300 valves were identified^30–35^ by the same analyst accredited within the UK Diatom Quality Assurance Scheme^36^. Calculation of EQR was undertaken using DARLEQ II software. More details are given in Supplementary Information.

### *In situ* environmental monitoring

Automatic weather stations installed in each catchment measured precipitation, air temperature, relative humidity and net solar radiation at 15 minute intervals^28^. A weather station centrally located between the three catchments produced total sunshine hours (Penrith Weather Station, 2017). *In situ* environmental and ecological monitoring occurred at catchment outflows (Fig. 1 (1.1)). Bank-side monitoring stations adjacent to biological sampling areas provided *in situ* water quality measurements at a minimum resolution of 15 minutes. Hach Lange nutrient analysers provided total reactive phosphorus (TRP) and nitrate (NO_3_-N) concentration data. Dissolved oxygen (electrochemical sensor, 0–500% ± 2%), pH (0–14 units ± 0.2 units), temperature (−5 to + 50 °C ± 0.15 °C), turbidity (0–1000 NTU, ±2% of reading or 0.3 NTU) and conductivity (0–100 µS cm^−1^ ± 0.5%) were analysed every 15 minutes using an YSI 6600 sonde. Discharge calculations were made by applying a stage-discharge relationship to 15 minute water level readings recorded by a pressure transducer. The stage-discharge relationship was developed through the collection of manual current metering measurements, and extrapolated beyond the gauged range using standard assumptions for the stage-discharge relationship and the hydrological water balance^37^. The *in situ* analysis was quality assured using monthly laboratory analysed samples to ensure accuracy in the measurements.

### Data analysis

PCA was used to determine the importance of environmental controls on the benthic diatom assemblages. PCA relates species composition to measured environmental gradients by reducing the dimensionality of complex, multivariate data, finding a small number of linear combinations of variables which are visualized using an ordination diagram^38^. All water chemistry data used within this analysis was averaged over the preceding 21 days to account for diatom community colonisation processes^20^. Values of turbidity, rainfall and discharge along with the species matrix were Log-transformed (Ax + B). All ordinations were performed using CANOCO version 5.1 software^39^.

Time-varying seasonal analysis (Mindham and Tych, 2018 *in submission)* of the data series was performed using ASDHR^40^ and conducted within Matlab. The approach belongs to the Unobserved Components Modelling group of methods applying spectral decomposition to the observation series ${y}_{t}$ isolating and estimating slow trends ${T}_{t}$ as well as cyclic and seasonal components ${C}_{t}$ and ${S}_{t}$ respectively and the irregular component ${e}_{t}$. This is executed using a stochastic state-space approach with Kalman Filtering and Fixed Interval Smoothing^40^ but placed within an irregular sampling framework. The arbitrary sampling, a novel approach in this context^41^, allows the Unobserved Components model to be estimated using irregularly sampled data.

$${y}_{t}={T}_{t}+{C}_{t}+{S}_{t}+{e}_{t}$$

In this case, no cyclic component was estimated and the seasonal component is expressed as:

$${S}_{t}=\sum _{i=1}^{{R}_{s}}\{{a}_{i,t}\,\cos ({\omega }_{i}t)+{b}_{i,t}\,\sin ({\omega }_{i}t)\}$$

where *a*_*i*,*t*_ and *b*_*i*,*t*_ are stochastic Time-Varying Parameters (TVP) and ${\omega }_{i}$ are the fundamental and harmonic frequencies associated with seasonality in the series (*i* = *1*, *2*, *…*, *R*_*s*_). The covariance parameters of the KF/FIS estimators are defined by the time scale of the estimated processes.

## Supplementary information

Supplementary Information

## Data Availability

Data can be obtained by contacting the corresponding author.
