# Supplementary material for: Strong and recurring seasonality revealed within stream diatom assemblages
Source: Sci Rep. 2019 Mar 1;9:3313. doi: 10.1038/s41598-018-37831-w (PMC6397146; doi:10.1038/s41598-018-37831-w)
Supplement: Supplementary file 1 — Supplementary Information [file 41598_2018_37831_MOESM1_ESM.docx]

**Supplementary Information**

**Strong and recurring seasonality revealed within stream diatom assemblages**

*M. A. SNELL^a,b^, P. A. BARKER^a^, B. W. J. SURRIDGE^a^, C. McW. H. BENSKIN^a^, N. BARBER^c^, S. M. REANEY^c^, W. TYCH^a^, D. MINDHAM^a^, A. R. G. LARGE^d^, S. BURKE^e^, P. M. HAYGARTH^a^

^a^Lancaster Environment Centre, Lancaster University, Lancaster LA1 4YQ, UK.

^b^Agri-Food and Biosciences Institute, Newforge Lane, Belfast BT9 5PQ, UK.

^c^Department of Geography, Durham University, Durham DH1 3LE, UK.

^d^School of Geography, Politics and Sociology, Newcastle University, Newcastle upon Tyne NE1 7RU, UK

^e^British Geological Survey, Environmental Science Centre, Nicker Hill, Keyworth, Nottingham NG12 5GG, UK.

**Methods**

**Diatom preparation and identification:**

For diatom analyses, sampling locations were selected from mid-reach within riffle zones typical of hydromorphological conditions. Five representative cobbles, approximately 64 - 128 mm in diameter (Wentworth, 1922) were randomly selected, based on colouration and feel, at each site. The cobbles were placed in a tray and removed to bankside where the upper surface area of each cobble was scraped using a hard bristle brush (Kelly et al., 1998). 150ml of diatom suspension was collected in sampling containers and returned to the laboratory.

In the laboratory, 50 ml of 30% (100 volume) hydrogen peroxide (H_2_O_2_) solution was added to 50ml of diatom suspension and heated on a hot-plate at 80°C (± 10 °C) for an average of 120 minutes to oxidize organic material. Samples were then removed from the hot-plate and beakers topped-up with distilled water. Following settling for a minimum of 24 hours, the supernatant was decanted. The beaker containing the diatom suspension was then re-filled with distilled water. This settling period was repeated three times, with a minimum of 24 hours between each settlement period (CEN, 2003).

Permanent slides were prepared from the digested diatom suspension and fixed using Naphrax, a diatom mountant with a refractive index of 1.73. 300 diatom valves were identified and counted along transects at 1000x magnification, under oil immersion, with a Zeiss Axioskop microscope (CEN, 2004). Valves were identified using standard floras (Krammer and Lange-Bertalot, 1991a, Krammer and Lange-Bertalot, 1991b, Krammer and Lange-Bertalot, 1991c, Krammer and Lange-Bertalot, 1986, Kelly, 2000, Hofmann et al., 2011) by the same analyst accredited within the UK Diatom Quality Assurance Scheme (Kelly, 2013).

**Diatom Metric Calculation:**

Two metrics were applied to the diatom data: Ecological Quality Ratio (EQR) and Diatom Ecological Guilds (EGs). Further information on diatom assemblage composition, including TDIv4 and bio-volume, can be found within Snell, 2014.

The EQR is a metric stipulated under the EU Water Framework Directive (2000/60/EC) to ensure comparability between different assessment methods in the determination of ecological quality. It is based on the ratio of the observed score for a given surface body against that expected under reference, or minimally disturbed, conditions. The ratio is expressed as numerical scale between 0 and 1, with values closer to 1 representing reference conditions or “high” ecological status. For UK diatom samples, the calculation of EQR is based on the predicted Trophic Diatom Index v4 (TDI4) for a given alkalinity and season against the observed score (WFD-UKTAG, 2014). The TDI is based on the sensitivity of diatom assemblage composition to inorganic phosphorus nutrient concentrations (Kelly and Whitton, 1995, Kelly, 1998, Kelly et al., 2001). Calculation of Ecological Quality Ratio (EQR) was undertaken using DARLEQ II software (Kelly, 2014).

Classifying the diatom taxa into EGs was based on three diatom growth morphologies: low profile, high profile and motile (Rimet and Bouchez, 2012). The low profile guild comprise species of short statures including prostrate (adhering to the substrate with the entire valve surface), adnate (apically attached but parallel to the substrate), erect (apically attached but perpendicular to the substrate) and slow-moving species such as *Achnanthes, Achnanthidium, Amphora, Cocconeis, Cymbella, Meridion* and *Reimeria*” species. These taxa are expected to be adapted to high current velocities and to low nutrient concentrations. The high-profile species were “species of tall stature including erect, filamentous, branched, chain-forming, tube forming, stalked, and colonial centrics from the following genera; *Diatoma, Ellerbeckia, Eunotia, Fragilaria, Gomphonema, Melosira (Melosira varians)* and *Synedra*”*.* Also, included in this guild are tube-forming diatoms, *Encyonema* and *Frustulia,* and filamentous benthic taxa such as *Melosira varians*. This guild is adapted to high nutrients concentrations and low current velocities. The motile guild includes “fast moving species” from the genera *Navicula, Nitzschia, Sellaphora* and *Surrirella*. This guild is sensitive to relatively high current velocities and high nutrient concentrations (Passy, 2007).

**References**

CEN 2003. 13946: Water quality-Guidance standard for the routine sampling and pretreatment of benthic diatoms from rivers. *TC.*

CEN 2004. Water Quality–Guidance Standard for the Identification, Enumeration and Interpretation of Benthic Diatom Samples from Running Waters. EN 14407: 2004. Comité Européen de Normalisation Geneva.

HOFMANN, G., WERUM, M. & LANGE-BERTALOT, H. 2011. *Diatomeen im Süßwasser-Benthos von Mitteleuropa: Bestimmungsflora Kieselalgen für die ökologische Praxis; über 700 der häufigsten Arten und ihrer Ökologie*, Gantner.

KELLY, M. 1998. Use of the trophic diatom index to monitor eutrophication in rivers. *Water research,* 32**,** 236-242.

KELLY, M. 2000. Identification of common benthic diatoms in rivers. *Field Stud,* 9**,** 583-700.

KELLY, M. 2013. Building capacity for ecological assessment using diatoms in UK rivers. *Journal of Ecology and Environment,* 36**,** 89-94.

KELLY, M., ADAMS, C., GRAVES, A., JAMIESON, J., KROKOWSKI, J., LYCETT, E., MURRAY-BLIGH, J., PRITCHARD, S. & WILKINS, C. 2001. The trophic diatom index: a user’s manual. *Revised Edition. Environment Agency, Bristol, BS32 4UD***,** 1-74.

KELLY, M., CAZAUBON, A., CORING, E., DELL'UOMO, A., ECTOR, L., GOLDSMITH, B., GUASCH, H., HÜRLIMANN, J., JARLMAN, A. & KAWECKA, B. 1998. Recommendations for the routine sampling of diatoms for water quality assessments in Europe. *Journal of applied Phycology,* 10**,** 215.

KELLY, M. & WHITTON, B. A. 1995. The trophic diatom index: a new index for monitoring eutrophication in rivers. *Journal of Applied Phycology,* 7**,** 433-444.

KELLY, M. G., JUGGINS, S.,BENNION, H., BURGESS, A., YALLOP, M., HIRST, H., JAMIESON, J., GUTHRIE, R., RIPPEY, B. 2014. DARLEQ: Diatom Assessment of River and Lake Ecological Quality Version 2.0.

KRAMMER, K. & LANGE-BERTALOT, H. 1986. Süsswasserflora von Mitteleuropa. Bacillariophyceae. 1. Teil: Naviculaceae, vol 2/1. *Book in German]. G. Fischer Verlag, Jena*.

KRAMMER, K. & LANGE-BERTALOT, H. 1991a. Süßwasserflora von Mitteleuropa, Bd 2/4. Bacillariophyceae. 4. Teil: Achnanthaceae Kritische Ergänzungen zu Navicula (Lineolatae) und Gomphonema. Gustav Fisher Verlag: Stuttgart.

KRAMMER, K. & LANGE-BERTALOT, H. 1991b. Susswasserflora von Mitteleuropa. Bacillariophyceae Teil iv: Achnanthaceae. Gustav Fischer Verlag: Stuttgart.

KRAMMER, K. & LANGE-BERTALOT, H. 1991c. Süßwasserflora von Mitteleuropa. Bacillariophyceae, Band 2/3, 3. Teil: Centrales, Fragillariaceae, Eunoticeae. 1-576. Stuttgart: Gustav Fischer Verlag.

PASSY, S. I. 2007. Diatom ecological guilds display distinct and predictable behavior along nutrient and disturbance gradients in running waters. *Aquatic Botany,* 86**,** 171-178.

RIMET, F. & BOUCHEZ, A. 2012. Life-forms, cell-sizes and ecological guilds of diatoms in European rivers. *Knowledge and management of Aquatic Ecosystems***,** 01.

SNELL, M. A. 2014. *Headwater Stream Biofilm Structure and Function at High Resolution Spatial-Temporal Scales.* PhD, Lancaster University

WENTWORTH, C. K. 1922. A scale of grade and class terms for clastic sediments. *The journal of geology,* 30**,** 377-392.

WFD-UKTAG 2014. River Assessment Method Macrophytes and Phytobenthos. Scotland: Water Framework Directive – United Kingdom Advisory Group
